# Socio-Demographic, Clinical and Psychological Profile of Frailty Patients Living in the Home Environment and Nursing Homes: A Cross-Sectional Study

**DOI:** 10.3389/fpsyt.2021.736804

**Published:** 2021-12-07

**Authors:** Marta Muszalik, Agnieszka Kotarba, Ewa Borowiak, Grażyna Puto, Mateusz Cybulski, Kornelia Kȩdziora-Kornatowska

**Affiliations:** ^1^Department of Geriatrics, Faculty of Health Sciences, Ludwik Rydygier Collegium Medicum in Bydgoszcz, Nicolaus Copernicus University in Toruń, Bydgoszcz, Poland; ^2^Department of Nursing Pedagogics, Faculty of Health Sciences, Medical University of Lodz, Lodz, Poland; ^3^Department of Conservative Nursing, Faculty of Health Sciences, Medical University of Lodz, Lodz, Poland; ^4^Department of Internal and Environmental Nursing, Institute of Nursing and Midwifery, Faculty of Health Sciences, Jagiellonian University Medical College, Kraków, Poland; ^5^Department of Integrated Medical Care, Faculty of Health Sciences, Medical University of Bialystok, Bialystok, Poland

**Keywords:** frailty syndrome, older patients, quality of life, cognitive and mental functioning, SHARE-FI, WHOQOL-BREF, GDS, MMSE

## Abstract

**Introduction:** Frailty syndrome, as a physiological syndrome, is characterized by a gradual decline in physiological reserve and a lowered resistance to stress-inducing factors, leading to an increased risk of adverse outcomes. It is significantly connected with dependence on care and frequent hospitalizations.

**Objectives:** The aim of the study was to describe socio-demographic, clinical and psychological profile of frailty older adults living in their own homes and to nursing homes.

**Methods:** The study was conducted with 180 patients who were over 60 years of age, the mean (±SD) was 74.1 (±8.8) years. Among the subjects, 90 individuals were community-dwelling older adults. The survey used a list of socio-demographic questions, as well as the following scales: Mini-Mental State Examination (MMSE), Geriatric Depression Scale (GDS), SHARE-FI, and The World Health Organization Quality of Life (WHOQOL-Bref).

**Results:** Pre-frailty was confirmed in 49 (27.2%) patients, and frailty syndrome was noticed in 47 patients (26.1%). The prevalence of frailty syndrome in the study group was related to: place of living (*p* < 0.001), age (*p* < 0.001), widowhood (*p* < 0.001), a poor economic situation (*p* < 0.001), basic education level (*p* < 0.001), living alone (*p* < 0.001), longer duration of illness (*p* < 0.001), comorbidities (*p* < 0.001), more medications taken (*p* < 0.001), deterioration of hearing (*p* = 0.003), impairment of cognitive functions (*p* < 0.001), depression (*p* < 0.001), and decreased quality of life (*p* < 0.001).

**Discussion:** A lot of socio-demographic and medical factors, particularly cognitive and mental functioning were connected with the prevalence and progression of frailty syndrome in the study group. Quality of life was significantly dependent on the presence of frailty syndrome, both in homes and in nursing homes.

## Introduction

Dynamic aging of societies remarkably increases the risk of frailty incidence in populations over 60 years of age (1, 2). Many factors are determining the occurrence of the frailty syndrome, e.g., age and other socio-demographic factors, physical and mental status, chronic diseases, medications, or place of living (home or nursing homes).

There are two different approaches to the assessment of frailty: one refers to frailty as a medical concept and defines frailty as a physical phenotype (3) and the second considers frailty more as a multidimensional concept, which not only refers to physical functioning, but also to psychological and social aspects (4). Fried et al., defines frailty syndrome as “a biologic syndrome of decreased reserve and resistance to stressors, resulting from cumulative declines across multiple physiologic systems, and causing vulnerability to adverse outcomes” (3). According to Fried et al. frailty syndrome is characterized by loss in body mass, reduced muscle strength, exhaustion, slowness, and low physical activity and can be confirmed if at least three out of the above-mentioned five criteria occur (3). According to Mitnitski et al. frailty is an age-associated, nonspecific vulnerability, in which the researchers considered symptoms, signs, diseases, and disabilities as deficits (4).

Santos-Eggimann et al. (5) considered Fried's criteria in the first European research regarding frailty [The Survey of Health, Aging and Retirement in Europe (SHARE)] in the group of middle-aged (above 50) and older community-dwelling Europeans living in ten countries (5). It was the first attempt at operationalizing Fried's frailty phenotype in a very large European population-based sample. The researchers distinguished five SHARE criteria based on clinical criteria according to Fried: weight loss, low physical activity, slow gait, decreased muscle strength, and exhaustion (5).

The condition may be diagnosed if at least three occur. The presence of one or two of these symptoms indicates the early stages of onset (pre-frailty) (3–5). More recent perspectives have described frailty in its broader multidimensional psychological and socio-demographical aspects (6–9).

The occurrence of frailty syndrome increases the risk of geriatric diseases, hospitalization, disability, loss of physical independence, and finally death. Therefore, prevention and early diagnosis are very important for the implementation of appropriate intervention measures that may minimize the negative effects of the condition, and improve older people's quality of life (10–13). Frailty prevention is a priority when planning health care for individuals who are 60 years of age or older (14–16). It is important to diagnose the occurrence of frailty and pre-frailty states as early as possible in individuals aged over 60 at the primary health care level (17).

Intervention management should be planned at the earliest opportunity in a patient's everyday life environment, with consideration given to adapting their living conditions and external environment to independent functioning (18). Effectively implemented prevention strategies at the primary health care level improve the quality of life of older adults, promotes healthy aging, and reduces the costs of health and social care systems (3, 19, 20). The aim of the study was to describe socio-demographic, clinical and psychological profile of frailty older adults living in their own homes and to nursing homes.

## Materials and Methods

### Participants

The study was conducted with 180 older patients of Polish nationality who were at least 60 years of age (as defined by the World Health Organization), the mean (±SD) were 74.1 (±8.8) years, respectively. Among the surveyed, 90 people were living in homes and participating in various forms of psychosocial activities as part of a senior club or community center. Most people (82.2%) had been participating in one type of activity (such as choir, dance, computer classes, and music therapy) once a week for at least 6 months. The second group consisted of 90 people living in nursing homes. All subjects had comorbidities. Among 180 (153 women and 27 men) older people included in the analysis (living in homes and nursing homes). The percentage of women was higher than the percentage of men (85 vs. 15%). The full socio-demographic and medical characteristics are presented in Table 1.

**Table 1:** Sociodemographic and clinical characteristics of the study group (*n* = 180).

**Characteristics**		**Total *n* = 180**	**Home environment *n* = 90**	**Nursing homes *n* = 90**	***p***
Age	Mean ± SD	74.1 ± 8.8	68.3 ± 3.5	79.9 ± 8.7	*p* < 0.001
Sex *n* (%)	men	27 (15)	10 (11.1)	17 (18.9)	NS
	women	153 (85)	80 (88.9)	73 (81.1)	
Marital status *n* (%)	married	62 (34.4)	46 (51.1)	16 (17.8)	*p* < 0.001
	not married/single	26 (14.4)	14 (15.6)	12 (13.3)	NS
	widowed	92 (51.1)	30 (33.3)	62 (68.9)	*p* < 0.001
Economic status *n* (%)	good	53 (29.4)	35 (38.9)	18 (20)	*p* = 0.009
	average	104 (57.8)	55 (611)	49 (54.4)	NS
	bad	23 (12.8)	0 (0.0)	23 (25.6)	*p* < 0.001
Education *n* (%)	primary	62 (34.4)	24 (26.7)	38 (42.2)	*p* = 0.04
	secondary	45 (25)	27 (30)	18 (20)	NS
	polytechnic	45 (25)	24 (26.7)	21 (23.3)	NS
	university	28 (15.6)	15 (16.6)	13 (14.5)	NS
Place of living *n* (%)	urban	159 (88.3)	74 (82.2)	85 (94.4)	*p* = 0.02
	rural	21 (11.7)	16 (17.8)	5 (5.6)	
Living status *n* (%)	alone	104 (57.8)	30 (33.3)	74 (82.2)	*p* < 0.001
	with family	76 (42.2)	60 (66.7)	16 (17.8)	*p* < 0.001
Number of diseases *n* (%)	Mean ± SD	2.3 ± 1.4	1.9 ± 0.8	2.7 ± 1.6	*p* < 0.001
	3 or more	69 (38.3)	19 (21.1)	50 (55.6)	*p* < 0.001
Duration of illness (years) *n* (%)	1–15	112 (62.2)	70 (77.8)	42 (467)	*p* < 0.001
	over 15	68 (37.8)	20 (22.2)	48 (53.3)	*p* < 0.001
Number of medicines *n* (%)	mean ± SD	4.9 ± 3.0	3.9 ± 2.1	6.0 ± 3.4	*p* < 0.001
	0	6 (3.3)	1 (1.1)	5 (5.6)	———
	1–5	120 (66.7)	75 (83.3)	45 (50)	*p* < 0.001
	6 or more	60 (33.3)	15 (16.7)	45 (50)	*p* < 0.001
Use of glasses					
*n* (%)		141 (78.3)	70 (77.8)	71 (78.9)	NS
Use of a hearing aid					
*n* (%)		19 (10.6)	4 (4.4)	15 (16.7)	*p* = 0.02

*n, number of respondents; %, percentage of respondents; SD, standard deviation; NS, not significant; p, p value for Student's t-test for independent samples; p, p value for comparison between residential homes and nursing homes for Pearson's chi2 test/Fisher's exact test, respectively*.

### Study Design and Data Collection

Patients living in homes and nursing homes, aged 60 and over, consent to participate in the study were the criteria for the inclusion in the study. The exclusion criteria (from both groups) were: aged under 60, communication difficulties, suffering from an exacerbation of comorbidities, recent stroke, severe oncological diseases, and lack of consent from the patient to participate in the study.

**Figure 1:**
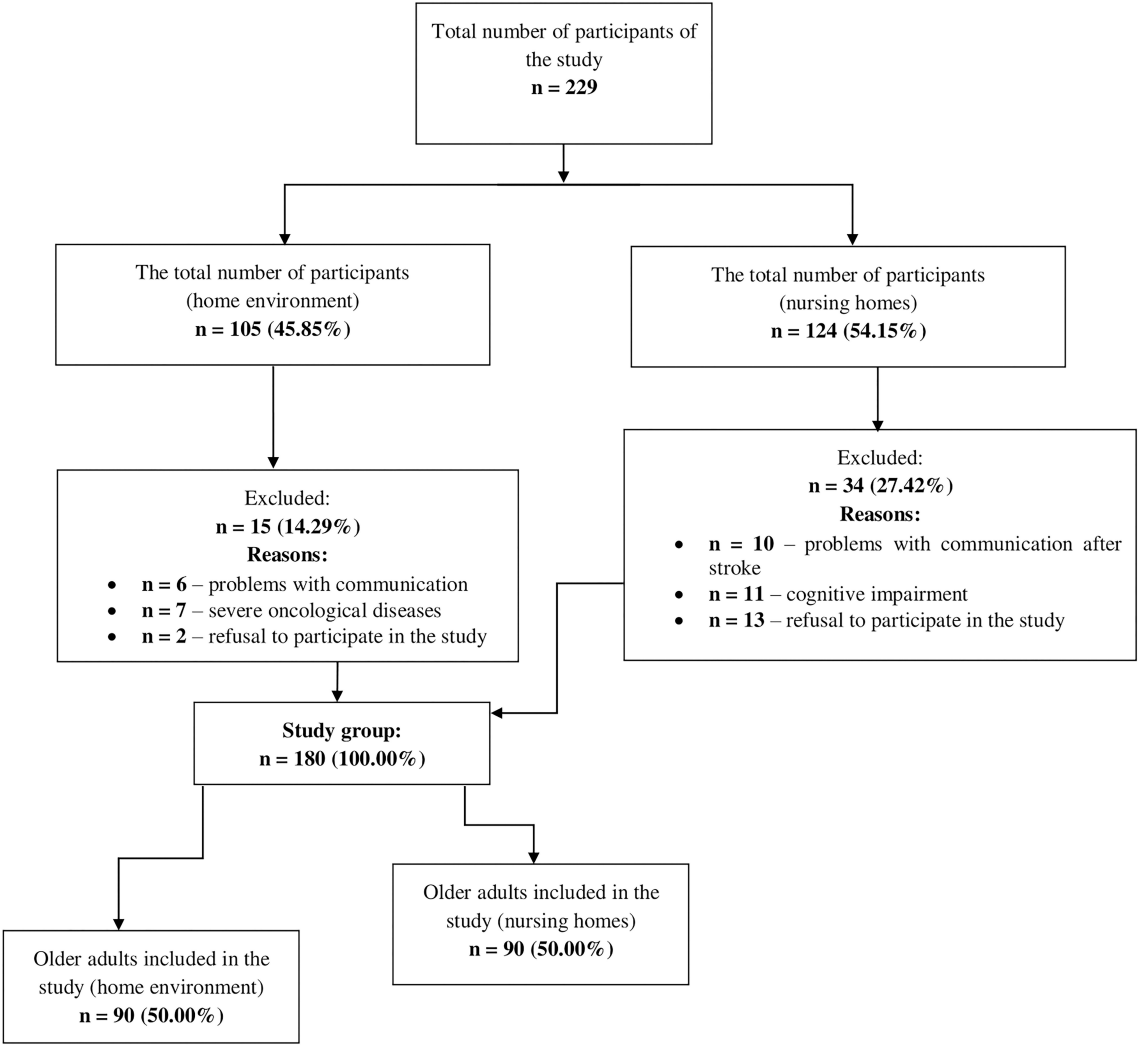
Patients enrolment in the study.

Each participant, before agreeing to participate in the study, was informed about its aims, how they would participate, the fact that the study would be anonymous, and the possibility of withdrawal from participation at any stage. The study was conducted using questionnaires that were prepared by a qualified nurse (members of the research team). The examination was voluntary and the sample selection was random. Figure 1 revealed the consort flow diagram of the study.

The research was carried out from October 2017 to October 2019 as part of the project “Adaptation and reliability testing of the SHARE-FI instrument for the assessment of the risk of frailty syndrome among older Polish patients.”

### Measures

We chose the most important and most frequently used standardized research tools in scientific research carried out among the older adults, which are characterized by high sensitivity and reliability and have been validated for Polish conditions.

#### The Frailty Instrument of the Survey of Health, Aging and Retirement in Europe

Frailty syndrome was assessed using the Frailty Instrument of the Survey of Health, Aging and Retirement in Europe (SHARE-FI), which is recommended as a screening test in primary health care and hospital care in people over 60 years of age of both sexes (3, 21). Translation and validation procedure of the Polish version of the SHARE-FI was completed by Muszalik (22). The questions included in the SHARE-FI concern the following areas: the subject's gender, subjective feeling of exhaustion, loss of appetite, difficulty in walking upstairs, reduction in physical activity, and assessment of handgrip strength (measured with a manual hydraulic dynamometer SAEHAN SH501) (22, 23). The obtained numerical values calculated with the use of the SHARE-FI virtual calculator allow the subject to be classified into one of the three groups: non-frail, pre-frail and frail (23). Qualification of the subject as a frail: score >3 for men and >2.13 for women; pre-frail: 1.21–3 for men and 0.32–2.13 for women and non-frail: <1.21 for men and <0.32 for women (5).

#### World Health Organization Quality of Life Questionnaire (WHOQOL)

Quality of life was assessed using the WHOQOL-Bref shortened Polish version of the World Health Organization Quality of Life Questionnaire (WHOQOL).

Based on the total assessment of the respondent's answers to 26 questions, an individual quality of life profile is established in the following categories:

– physical (Domain 1): including the level of demand for medical treatment, general fitness, ability to perform daily activities;
– psychological (Domain 2): self-esteem in everyday life, including satisfaction with an individual's appearance.
– social relations (Domain 3): relationships and interpersonal relationships, sexual activity, social support.
– functioning in the environment (Domain 4): financial resources, physical and mental security, health care (availability and quality), housing conditions, communication/transport (24).

The guidelines of the World Health Organization (WHO) for the conversion of the raw values obtained for individual domains into a score ranging from 0 to 100 points were applied. As a result, the results can be compared with the WHOQOL-100 version. Additionally, the questionnaire contains two questions that are analyzed separately: a general perception of the overall quality of life, and a general self-assessment of health. Answers are given on a 5-point scale (score range 1–5) (24).

The results of individual domains of the general perception of the quality of life and self-assessment of health are positive (i.e., the greater the number of points, the higher the quality of life) (24).

#### Mini-Mental State Examination (MMSE)

Cognitive performance was evaluated using the Polish version of the Mini-Mental State Examination (MMSE) scale. The scale consists of 30 questions which allow for the quantitative assessment of different aspects of cognitive functioning. The areas subject to evaluation are as follows: orientation to time and place, registration, attention and calculation, recall, language, repetition, reading comprehension, writing, and drawing. The maximum score is 30 points. The cut-off point of 23 is a sensitive indicator of cognitive decline and indicates the need for specialized testing. The obtained score can also be related to a specific category: 27–30 is a normal result, 24–26 signifies cognitive disorders without dementia, 19–23 suggests mild dementia, 11–18 denotes moderate dementia, while 0–10 reflects deep dementia (25).

#### Geriatric Depression Scale (GDS)

Depression was assessed using a 15-point Geriatric Depression Scale (GDS). This is a screening tool that is used to evaluate the severity of depression symptoms in older people. The interpretation of results is as follows: 0 to 5 points mean healthy condition, 6 to 10 points signifies a moderate sense of depression, and 11 to 15 points signify a deep sense of depression (26).

### Procedure and Ethical Considerations

The study was carried out in accordance with the recommendations, and was reviewed and approved by the Bioethics Committee of the Medical University of Lodz (statute no. RNN/185/16/KE). All subjects gave the written informed consent in accordance with the Declaration of Helsinki.

### Statistical Analysis

An analysis of qualitative variables was described using absolute numbers in particular categories (n), and their percentage participation in the distribution of the variables (%). Average values of variables with a normal distribution were described by their mean and standard deviation (SD). An analysis of the group comparison between quantitative variables was conducted with the application of a chi-squared test if the expected values in at least 80% of all cells of the cross table representing a particular relationship were higher than 5. Otherwise, for 2 × 2 table sizes, Fisher's exact test was applied, with a chi-squared exact test for tables of other sizes. Normal distribution of quantitative variables was checked with Shapiro–Wilk W-test. As none of the variables was distributed normally, they were analyzed with non-parametric tests. An analysis of the group comparison between respondents' age and quantitative variables was conducted using Student's *t*-test for independent groups and in the case of other quantitative variables using the Mann-Whitney test. An analysis of the group comparison between respondents' age and SHARE-FI was conducted using ANOVA and in the case of other quantitative variables using the Kruskal-Wallis test. A statistical analysis of the findings was carried out with IBM SPSS Statistics 24 for Windows (IBM Corp., Armonk, NY, USA). In all conducted analyses, the differences in the intensity and strength of the relationships between variables were assessed with the significance level *p* < 0.05.

## Results

Prevalence of frailty and pre-frail in the nursing home was 47.8 and 41.1% respectively. The prevalence of frailty syndrome and pre-frailty syndrome was statistically significantly more frequently (*p* < 0.001) in the inhabitants of nursing homes than in people living in own homes. The results are presented in Table 2.

**Table 2:** Characteristics of frailty syndrome according to the SHARE-FI in the study group (*n* = 180).

**Characteristics**	**Total *n* = 180**	**Home environment *n* = 90**	**Nursing homes *n* = 90**	***p***
Frailty score (mean ± SD)	0.4 ± 2.3	−1.4 ± 1.7	2.2 ± 1.3	*p* < 0.001
Frail *n* (%)	47 (26.1)	4 (4.5)	43 (47.8)	*p* < 0.001
Pre-frail *n* (%)	49 (27.2)	12 (13.3)	37 (41.1)	*p* < 0.001
Non-frail *n* (%)	84 (46.7)	74 (82.2)	10 (11.1)	*p* < 0.001

*n, number of respondents; %, percentage of respondents; P-value for the Mann-Whitney test*.

Among the people diagnosed with frailty syndrome based on the SHARE-FI scale, the mean age was 80.7 years (±9.0) than in the pre-frail group – 76.7 years (±8.5), or non-frail – 69.0 years (±5.0) (*p* < 0.001). There were no significant differences between the sex of the subjects and the frequency of frailty syndrome occurrence. People with frailty were significantly more often widowed and lived alone, while non-frail patients were married (*p* < 0.001). People with frailty syndrome had a statistically significantly higher number of diseases, medications and were ill longer compared to patients with pre-frail and no-frail (*p* < 0.001). A greater number of depressive symptoms and lower level of cognitive function were demonstrated by patients with frailty compared to the pre-frail group (*p* < 0.001). Patients with frailty syndrome had a lower general quality of life. The results are presented in Table 3.

**Table 3:** Sociodemographic and clinical characteristics according to SHARE-FI in the study group (*n* = 180).

**Characteristics**		**SHARE-FI**			***p***
		**Non-Frail *n* (%) 84 (46.7)**	**Pre-Frail *n* (%) 49 (27.2)**	**Frail *n* (%) 47 (26.1)**	
Age	Mean ± SD	69.0 ± 5.0^*^^†^	76.7 ± 8.5^†^	80.7 ± 9.0^*^	p <0.001
Sex *n* (%)	men	13 (15.5)	10 (20.4)	4 (8.5)	NS
	women	71 (84.5)	39 (79.6)	43 (915)	
Marital status *n* (%)	married	44 (52.4)	9 (18.4)	9 (19.2)	*p* < 0.001
	not married/single	17 (20.2)	4 (8.2)	5 (10.6)	NS
	widowed	23 (27.4)	36 (73.4)	33 (70.2)	*p* < 0.001
Economic status *n* (%)	good	36 (42.9)	7 (14.3)	10 (21.3)	*p* < 0.001
	average	48 (57.1)	30 (61.2)	26 (55.3)	NS
	bad	0 (0.0)	12 (24.5)	11(23.4)	*p* < 0.001
Education *n* (%)	primary	16 (19.1)	27(55.1)	19 (40.4)	*p* < 0.001
	secondary	21 (25.0)	12 (24.5)	12 (25.5)	NS
	polytechnic	28 (33.3)	8 (16.3)	9 (19.2)	NS
	university	19 (22.6)	2 (4.1)	7 (14.9)	*p* = 0.02
Living status *n* (%)	alone	33 (39.3)	37 (75.5)	34 (72.3)	*p* < 0.001
	with family	51 (60.7)	12 (24.5)	13 (27.7)	*p* < 0.001
Duration of illness (years) *n* (%)	1–15	67 (79.8)	27 (55.1)	18 (38.3)	*p* < 0.001
	Over 15	17 (20.2)	22 (44.9)	29 (61.7)	*p* < 0.001
Number of diseases (mean ± SD)		1.9 ± 0.9^*^^†^	2.6 ± 1.4	2.9 ± 1.8	*p* < 0.001
Number of medicines (mean ± SD)		3.5 ± 1.9^*^^†^	5.6 ± 2.7^†^	6.7 ± 3.5^*^	*p* < 0.001
Use of glasses					
*n* (%)		4 (3.8)	4 (6.2)	10 (2.4)	NS
Use of a hearing aid					
*n* (%)		4 (4.8)	4 (8.2)	11 (23.4)	*p* = 0.003
MMSE	mean ± SD	27.6 ± 2.3^*^^†^	24.6 ± 4.0	23.9 ± 4.5	*p* < 0.001
	24–26 n (%)	5 (6.0)	15 (30.6)	15 (31.9)	*p* < 0.001
	27–30 n (%)	79 (94.0)	34 (69.4)	32 (68.1)	*p* < 0.001
GDS	mean ± SD	2.4 ± 2.3^*^^†^	5.4 ± 2.9	6.2 ± 2.8	*p* < 0.001
	0–5 n (%)	76 (90.5)	29 (59.2)	20 (42.6)	*p* < 0.001
	6–15 n (%)	8 (9.5)	20 (40.8)	27 (57.4)	*p* < 0.001
WHOQOL-BREF—Environment (0–100)					
mean ± SD		16.5 ± 5.2 ^*^^†^	23.0 ± 7.5	25.2 ± 5.5	*p* < 0.001
Overall quality of life (1–5)					
mean ± SD		4.0 ± 0.7 ^*^^†^	3.3 ± 0.7	3.3 ± 0.9	*p* < 0.001
General health (1–5)					
mean ± SD		3.6 ± 0.7 ^*^^†^	3.1 ± 1.0	2.7 ± 0.9	*p* < 0.001

*n, number of respondents; %, percentage of respondents; SD, standard deviation; NS, not significant; p, p value for Student's t-test for independent samples; p, p value for comparison between residential homes and nursing homes for Pearson's chi2 test/Fisher's exact test, respectively*.

* *Statistical significance (p <0.05) as compared to the pre-frail group*.

† *Statistical significance (p <0.05) as compared to the frail group*.

The subjective assessment of the quality of life carried out with the WHOQOL-Bref questionnaire was significantly higher among people living in homes than among those living in nursing homes. Patients from nursing homes had significantly higher physical, psychological, and environmental scores than in homes (*p* < 0.001). Social relationship scores were higher in homes than in nursing homes (*p* < 0.001). The overall perception of quality of life and the general self-assessment of health were significantly higher in homes than in nursing homes. Cognitive performance was assessed by the Mini Mental State Examination and it showed a lower mean score in people with frailty syndrome (23.9 ± 4.5) than in those in the pre-frail group (24 ± 4.0) and in the non-frail group (27.6 ± 2.3). The results are presented in Table 4.

**Table 4:** Quality of life assessment carried out with the WHOQOL-Bref questionnaire in the study group (*n* = 180).

**Domains**	**Total *n* = 180**	**Home environment *n* = 90**	**Nursing homes *n* = 90**	***p***
Physical health (0–100) mean ± SD	36.1 ± 17.2	22.9 ± 5.6	49.3 ± 14.5	*p* < 0.001
Psychological (0–100) mean ± SD	45.7 ± 16.4	35.1 ± 7.4	56.3 ± 16.1	*p* < 0.001
Social relationships (0–100) mean ± SD	74.0 ± 25.1	91.1 ± 11.5	56.9 ± 23.4	*p* < 0.001
Environment (0–100) mean ± SD	40.9 ± 22.1	21.9 ± 5.8	59.8 ± 15	*p* < 0.001
Overall quality of life (1–5) mean ± SD	3.6 ± 0.8	3.8 ± 0.7	3.4 ± 0.9	*p* < 0.001
General health (1–5) mean ± SD	3.3 ± 0.9	3.5 ± 0.8	3.0 ± 0.9	*p* < 0.001

*n, number of respondents; %, percentage of respondents; SD, standard deviation; p-value for the Mann-Whitney test*.

People living in nursing home were significantly older (*p* < 0.001), had more diseases (*p* < 0.001) and had significantly more weakness syndrome (*p* < 0.001) than those living at home. Due to the above, they also had a significantly lower quality of life.

## Discussion

Frailty syndrome has acquired particular importance, not only because of the deregulation of many systems and organs that progresses with age, but also because of the consequences of the increased risk of disability, reliance on the care of other people, and institutionalization. The results of this study, conducted with 180 people over 60 years of age, showed that the prevalence of pre-frailty syndrome was 27.2% and the level of actual suffering from frailty syndrome was 26.1%. People in nursing homes showed frailer (47.8%) or pre-frail (41.1%) symptoms than those who lived in homes. The available data in the literature are in line with the results obtained in the authors' research, and prove that frailty syndrome is a widespread problem of the aging population in Poland, as well as in the rest of the world (27). In Europe, the highest average results were recorded in Italy, Spain, and Poland, and the lowest in Denmark, Switzerland, and Ireland (28). A study by Liu et al. confirmed the prevalence of frailty syndrome among people over 60 years of age living in nursing homes. This study showed that the age of women is significantly correlated with the occurrence of frailty syndrome (29). In our study, no relationship was found between the occurrence of frailty syndrome and the gender of the respondents, while an increase in its occurrence with age was confirmed. Being female as a factor predisposing one to frailty syndrome was also demonstrated in a study by Shibasaki et al. (30). A study by Xu et al. in China on people over 60 showed that the incidence of frailty syndrome was 60.6 per 1,000 person-years and increased with age, more often in women than in men (27). Women are more prone to frailty because they live longer and accumulate more weaknesses, and tend to have lower body weight and muscle strength than men (31). Suffering from frailty in the conducted study was determined by marital status and the structure of the residence. Those who were widowed and living alone were characterized by frailty or pre-frailty, while those in the non-frail group were living with family and were married. Living alone or with other people (unknown person) in nursing homes, lack of regular exercise, and poor health were significantly correlated with the onset of frailty syndrome in a study by Liu et al. (29). Lower education and economic status, as well as suffering from chronic diseases, were significantly associated with the incidence of frailty syndrome in the study by Xu et al. (27). The present study confirmed that people in the pre-frail and frail groups showed a lower level of education and economic status.

In a study conducted by Saum et al. in Germany with 11 years of follow-up of the subjects, it was confirmed that multi-drug use results in a 1.5-fold increase of the risk of frailty syndrome within 3 years, notwithstanding the number of comorbidities and their severity (32). It is associated with an increased frequency of falls (33) and hospitalization, disability, and mortality (34, 35). The relationship between the number of comorbidities and their severity and the development of frailty syndrome was also confirmed in other subjects (35). The present study confirmed the relationship between the incidence of frailty syndrome and a greater mean number of diseases, represented by the medications taken, and the duration of the diseases. Cognitive impairment and depression symptoms have been shown in a study conducted in Turkey by Hammami et al. (36) to be a factor that increases the occurrence of frailty syndrome. A study by Lenardt et al. showed that physical disability, loss of independence, and increased frequency of hospitalization and institutionalization are all consequences of frailty syndrome that reduce the quality of life of older people (37). The relationship between quality of life and frailty syndrome has also been confirmed in people living with their families, where it has been shown that interventions designed to reduce frailty can have the additional benefit of improving quality of life. This study conducted a meta-analysis of 11 cross-sectional studies which confirmed that aggravation of frailty syndrome results in a reduction in the quality of life (11). The results of the meta-analysis are consistent with the results of our research, showing a relationship between a lower quality of life score and the progression of frailty syndrome. People living in nursing homes showed a greater progression of frailty syndrome and a lower quality of life score than those living with their families. The relationship between the severity of frailty syndrome and both qualities of life and physical wellbeing was also confirmed in another study with the use of the WHOQOL-BREF questionnaire (38).

### Limitations

The number of older adults participating in the study is the first limitation. The study was single-center in a group of 180 patients. The small sample size does not allow for extrapolation of results to the general population of older adults in Poland. Despite the quantitative limitation, the results of the study were confirmed by statistical methods. Significant differences were noticed between the studied groups. Taking into account population aging, there is a need for long-term monitoring of the risk factors of frailty syndrome to shape the policy of health services and support and care services. The aim should be primarily to obtain information that can be applied to individual older people in their particular conditions while taking into account the limitations resulting from the existing frailty syndrome. The inclusion of the results of the research on frailty syndrome in the daily practice of the therapeutic team should be the basis for cooperation with older adults. Thus, it could create the possibility of redefining the concept of therapeutic success from a purely biological dimension to the bio-psycho-social dimensions. However, this calls for several systemic changes, both social and at the level of education of the therapeutic team, as well as in the system used for financing health benefits for people this age. These changes will become possible only through further attempts at analysis in this field. The research results presented here may serve as an indicator for further exploration, which undoubtedly should continue with ever greater precision.

In conclusion, the prevalence and progression factors of frailty syndrome in the study were connected with age, marital status, economic status, level of education, the structure of the residence, duration of illness, number of comorbidities, number of medications taken, cognitive functioning, and depression. Quality of life was significantly conditioned by the presence of frailty syndrome, both in- home environments and in nursing homes. Multifactor interdisciplinary interventions in primary health care focused on identifying the risk factors of frailty syndrome should be a public health priority to shape the policy of health services, as well as that of support and care services. Raising the awareness of older people around healthy aging, including self-motivation to treat their ailments, should be a top priority to reduce morbidity and improve quality of life.

## Data Availability Statement

The original contributions presented in the study are included in the article/supplementary material, further inquiries can be directed to the corresponding author.

## Ethics Statement

The studies involving human participants were reviewed and approved by Bioethics Committee of the Medical University of Lodz (RNN/185/16/KE). The patients/participants provided their written informed consent to participate in this study.

## Author Contributions

AK, MM, MC, and EB: study design. AK and EB: data collection. AK, EB, and GP: data analysis. AK, EB, MM, MC, KK-K, and GP: data interpretation. AK, EB, MM, MC, and GP: preparation of manuscript. AK, EB, MM, MC, KK-K, and GP: literature analysis. AK and EB: funds collection. All authors critically revised the manuscript and agreed to be fully accountable for ensuring the integrity and accuracy of the work. All authors have read and agreed to the published version of the manuscript.

## Funding

The research was funded with grant no. WN 767 of the Nicolaus Copernicus University in Toruń, Ludwik Rydygier Collegium Medicum in Bydgoszcz.

## Conflict of Interest

The authors declare that the research was conducted in the absence of any commercial or financial relationships that could be construed as a potential conflict of interest.

## Publisher's Note

All claims expressed in this article are solely those of the authors and do not necessarily represent those of their affiliated organizations, or those of the publisher, the editors and the reviewers. Any product that may be evaluated in this article, or claim that may be made by its manufacturer, is not guaranteed or endorsed by the publisher.

## References

[1] Yon Y Mikton CR Gassoumis ZD Wilber KH. Elder abuse prevalence in community settings: a systematic review and meta-analysis. Lancet Glob Health. (2017) 5:e147–56. 10.1016/S2214-109X(17)30006-2 28104184

[2] Piejko L Nawrat-Szołtysik A. Treatment options for frailty syndrome in the elderly. Geriatrics. (2017) 11:283–9.

[3] Fried L Tangen C. Walston J Newman AB Hirsch C Gottdiener J. Frailty in older adults: evidence for a phenotype. J Gerontol Ser A Biol Sci Med Sci. (2001) 56:M146–56. 10.1093/gerona/56.3.M146 11253156

[4] Mitnitski AB Mogilner AJ Rockwood K. Accumulation of deficits as a proxy measure of aging. Sci World J. (2001) 1:323–36. 10.1100/tsw.2001.58 12806071 PMC6084020

[5] Santos-Eggimann B Cuenoud P Spagnoli J Junod J. Prevalence of frailty in middle-aged and older community-dwelling Europeans living in 10 countries. J Gerontol A Biol Sci Med Sci. (2009) 64:675–81. 10.1093/gerona/glp012 19276189 PMC2800805

[6] Xue QL. The frailty syndrome: definition and natural history. Clin Geriatr Med. (2011) 27:1–15. 10.1016/j.cger.2010.08.009 21093718 PMC3028599

[7] Puts MTE Toubasi S Andrew MK Ashe MC Ploeg J Atkinson E. Interventions to prevent or reduce the level of frailty in community-dwelling older adults: a scoping review of the literature and international policies. Age Ageing. (2017) 46:383–92. 10.1093/ageing/afw247 28064173 PMC5405756

[8] Bagshaw SM Stelfox HT Johnson JA McDermid RC Rolfson DB Tsuyuki RT. Long-term association between frailty and health-related quality of life among survivors of critical illness. Crit Care Med. (2015) 43:973–82. 10.1097/CCM.0000000000000860 25668751

[9] Gobbens RJ. Cross-sectional and longitudinal associations of environmental factors with frailty and disability in older people. Arch Gerontol Geriatr. (2019) 85:103901. 10.1016/j.archger.2019.103901 31352186

[10] Sutton JL Gould RL Daley S Coulson MC Ward EV Butler AM. Psychometric properties of multicomponent tools designed to assess frailty in older adults: a systematic review. BMC Geriatr. (2016) 16:1–20. 10.1186/s12877-016-0225-2 26927924 PMC4772336

[11] Kojima G Iliffe S Jivraj S Walters K. Association between frailty and quality of life among community-dwelling older people: a systematic review and meta-analysis. J Epidemiol Commun Health. (2016) 70:716–21. 10.1136/jech-2015-206717 26783304

[12] Darvall JN Boonstra T Norman J Murphy D Bailey M Iwashyna TJ. Retrospective frailty determination in critical illness from a review of the intensive care unit clinical record. Anaesth Intensive Care. (2019) 47:343–8. 10.1177/0310057X19856895 31342763

[13] Amer M El Akkad RM Hassan HS. Correlation of frailty status to health-related quality of life in the elderly: a cross-sectional study on community-dwelling older adults referred to an outpatient geriatric service in Egypt. Middle East J Age Ageing. (2015) 12:3–12. 10.5742/MEAA.2015.92608

[14] Buckinx F Rolland Y Reginster JY Ricour C Petermans J Bruyère O. Burden of frailty in the elderly population: perspectives for a public health challenge. Arch Public Health. (2015) 73:1–7. 10.1186/s13690-015-0068-x 25866625 PMC4392630

[15] Liotta G Ussai S Illario M O'Caoimh R Cano A Holland C. Frailty as the future core business of public health: report of the activities of the A3 action group of the European Innovation Partnership on Active and Healthy Ageing (EIP on AHA). Int J Environ Res Public Health. (2018) 15:2843. 10.3390/ijerph15122843 30551599 PMC6313423

[16] Marcucci M Damanti S Germini F Apostolo J Bobrowicz-Campos E Gwyther H. Interventions to prevent, delay or reverse frailty in older people: a journey towards clinical guidelines. BMC Med. (2019) 17:193. 10.1186/s12916-019-1434-2 31660959 PMC6819620

[17] Callaghan S Smith SM. Frailty in family practice. Fam Pract. (2017) 34:508–10. 10.1093/fampra/cmx029 28472329

[18] Piotrowicz J Soll A Kielar U Zwiefka A Guligowska A Pigłowska M. ICT and environmental support for patients with frailty syndrome: carewell project, focus project, and SUNFRAIL project. High Sch Pulse. (2017) 11:37–43. 10.5604/01.3001.0009.9274

[19] Sacha J Sacha M Soboń J Borysiuk Z Feusette P. Is it time to begin a public campaign concerning frailty and pre-frailty? A Review Article. Front Physiol. (2017) 8:484. 10.3389/fphys.2017.00484 28744225 PMC5504234

[20] Walters K Frost R Kharicha K Avgerinou C Gardner B Ricciardi F. Home-based health promotion for older people with mild frailty: The HomeHealth intervention development and feasibility RCT. Health Technol Assess. (2017) 21:1–128. 10.3310/hta21730 29214975 PMC5742456

[21] Romero-Ortuno R Walsh CD Lawlor BA Kenny RA, A. Frailty Instrument for primary care: findings from the Survey of Health, Ageing, and Retirement in Europe (SHARE). BMC Geriatr. (2010) 10:1–12. 10.1186/1471-2318-10-57 20731877 PMC2939541

[22] Muszalik M Borowiak E Kotarba A Puto G Doroszkiewicz H Kedziora-Kornatowska K. Adaptation and reliability testing of the SHARE-FI instrument for the assessment of risk of frailty syndrome among older polish patients. Fam Med Prim Care Rev. (2018) 20:36–40. 10.5114/fmpcr.2018.73702

[23] SHARE-FI, Calculator Females v,. 1.0. Available online at: https://sites.google.com/a/tcd.ie/share-frailty-instrument-calculators/translated-calculators (accessed October 19, 2021).

[24] Jaracz K Kalfoss M Górna K Baczyk G. Quality of life in polish respondents: psychometric properties of the polish WHOQOL—Bref. Scand J Caring Sci. (2006) 20:251–60. 10.1111/j.1471-6712.2006.00401.x 16922978

[25] Folstein MF Folstein SE McHugh PR. Mini-mental state. A practical method for grading the cognitive state of patients for the clinician. J Psychiatr Res. (1975) 12:189–98. 10.1016/0022-3956(75)90026-6 1202204

[26] Yesavage JA Brink TL Rose TL Lum O Huang V Adey M. Development and validation of a geriatric depression screening scale: a preliminary report. J Psychiatr Res. (1982) 17:37–49. 10.1016/0022-3956(82)90033-4 7183759

[27] Xu W Li YX Wu C. Incidence of frailty among community-dwelling older adults: A nationally representative profile in China. BMC Geriatr. (2019) 19:1–9. 10.1186/s12877-019-1393-7 31888498 PMC6937935

[28] Kurpas D Szwamel K Soll A. Bujnowska-Fedak, MM. Frailty syndrome — Guidelines for diagnosis therapy and prevention. Med Rodz. (2017) 25:6–13.

[29] Liu W Puts M Jiang F Zhou C Tang S Chen S. Physical frailty and its associated factors among elderly nursing home residents in China. BMC Geriatr. (2020) 20:1–9. 10.1186/s12877-020-01695-5 32807098 PMC7433121

[30] Shibasaki K Kin SK Yamada S Akishita M Ogawa S. Sex-related differences in the association between frailty and dietary consumption in Japanese older people: a cross-sectional study. BMC Geriatr. (2019) 19:1–9. 10.1186/s12877-019-1229-5 31382881 PMC6683375

[31] HeBMaYWangCJiangMGengCChangX. Prevalence and risk factors for frailty among community-dwelling older people in China: a systematic review and meta-analysis. J Nutr Health Aging. (2019) 23:442–50. 10.1007/s12603-019-1179-931021361

[32] Saum KU Schöttker B Meid AD Holleczek B Haefeli WE Hauer K. Is polypharmacy associated with frailty in older people? Results From the ESTHER Cohort Study. J Am Geriatr Soc. (2016) 65:e27–32. 10.1111/jgs.14718 28024089

[33] Bonaga B Sánchez-Jurado PM Martínez-Reig M Ariza G Rodríguez-Mañas L Gnjidic D. Frailty, polypharmacy, and health outcomes in older adults: the frailty and dependence in albacete study. J Am Med Dir Assoc. (2018) 19:46–52. 10.1016/j.jamda.2017.07.008 28899661

[34] Dhalwani NN Fahami R Sathanapally H Seidu S Davies MJ Khunti K. Association between polypharmacy and falls in older adults: A longitudinal study from England. BMJ Open. (2017) 7:e016358. 10.1136/bmjopen-2017-016358 29042378 PMC5652576

[35] Oude Voshaar RC Jeuring HW Borges MK van den Brink RHS Marijnissen RM. Hoogendijk EO. Course of frailty stratified by physical and mental multimorbidity patterns: a 5-year follow-up of 92,640 participants of the LifeLines cohort study. BMC Med. (2021) 19:29. 10.1186/s12916-021-01904-x 33550989 PMC7869455

[36] Hammami S Ghzaiel I Hammouda S Sakly N Hammami M Zarrouk A. Evaluation of pro-inflammatory cytokines in frail Tunisian older adults. PLoS ONE. (2020) 15:e0242152. 10.1371/journal.pone.0242152 33166358 PMC7652286

[37] Lenardt MH Carneiro NH Binotto MA Willig MH Lourenço TM Albino J. Frailty and quality of life in elderly primary health care users. Rev Bras Enferm. (2016) 69:478–83. 10.1590/0034-7167.2016690309i 27355296

[38] de Labra C Maseda A Lorenzo-López L López-López R Buján A Rodríguez-Villamil JL. Social factors and quality of life aspects on frailty syndrome in community-dwelling older adults: The VERISAÚDE study. BMC Geriatr. (2018) 18:1–9. 10.1186/s12877-018-0757-8 29514599 PMC5842614

